# Grhl2 reduces invasion and migration through inhibition of TGFβ-induced EMT in gastric cancer

**DOI:** 10.1038/oncsis.2016.83

**Published:** 2017-01-09

**Authors:** J Xiang, X Fu, W Ran, Z Wang

**Affiliations:** 1 Department of Gastrointestinal Surgery, The First Affiliated Hospital of Chongqing Medical University, Chongqing, People's Republic of China; 2 Department of General Surgery, Chongqing General Hospital, Chongqing, People's Republic of China; 3 Department of General Surgery, Qianjiang Central Hospital of Chongqing, Chongqing, People's Republic of China

## Abstract

Metastasis is one of the typical features of malignancy that significantly increases cancer-related mortality. Recent studies have shown that epithelial–mesenchymal transition (EMT) is closely related to the invasion and migration of cancer cells. Grainyhead-like 2 (Grhl2), a transcription factor, has been reported to be associated with several tumor processes including EMT. In the previous study, we have reported that Grhl2 functioned as a tumor suppressor in proliferation and apoptosis of gastric cancer. Here we aim to explore the effects of Grhl2 on invasion and migration of gastric cancer and further clarify its possible underlying mechanisms. As a result, in both SGC7901 and MKN45 cells, Grhl2 overexpression significantly inhibited the ability of invasion and migration. In addition, preliminary experiments showed that Grhl2 reduces the protein expression of matrix metalloproteinase-2, -7 and -9 (MMP-2, MMP-7 and MMP-9). Most importantly, Grhl2 antagonizes transforming growth factor-β (TGFβ)-induced EMT, and inhibition of TGFβ signaling pathways can restore Grhl2 expression. Finally, the results of subcutaneous xenograft model indicated that Grhl2 suppresses the growth of gastric cancer and reverses EMT process *in vivo*. Meanwhile, the metastatic tumor model further confirmed the inhibition of Grhl2 on metastasis of gastric cancer. Taken together, our findings proved that Grhl2, functioned as a tumor suppressor, reduces the invasion and migration through inhibition of TGFβ-induced EMT in gastric cancer.

## Introduction

Gastric cancer (GC) continues to be an important health threat as the third leading cause of cancer-related death in both sexes worldwide.^1^ However, most cancer patients do not die because of the primary cancer, but because of the metastatic cancer.^2^ Metastasis is one of the typical features of malignancy that significantly increases cancer-related mortality. Although research on metastasis has made some progress in recent years, the exact molecular mechanisms still need further study.

Epithelial-–mesenchymal transition (EMT) is a highly conserved developmental program that allows polarized, immotile epithelial cells to convert to those with motile mesenchymal properties.^3^ Actually, EMT is a physiological process during embryonic development that has crucial roles in the formation of the body plan and in the differentiation of multiple tissues and organs. In addition, EMT contributes to tissue repair.^4^ Meanwhile, growing evidence indicates that EMT is a phenotypic conversion linked to metastasis.^4, 5^ Research on the mechanisms of EMT has aroused great research interest, expecting to find a new breakthrough for the treatment and prevention of cancer metastasis. As far as we know, EMT is a complex pathophysiological process that can be triggered by a variety of soluble factors including epidermal growth factor, hepatocyte growth factor, fibroblast growth factor and transforming growth factor-β (TGFβ). Other stimuli such as hypoxia and adhesion to ECM components can also induce EMT.^6^ Among these, TGFβ family signaling has a predominant role.^7^

Grainyhead-like 2 (Grhl2), a transcription factor that belongs to the grainyhead-like(Grhl) family, has an important role in the establishment of epithelial polarity and for the acquisition and maintenance of epithelial-specific functions.^8^ Moreover, reduced Grhl2 expression affects the migration pattern of lung epithelial cells,^9^ and Grhl2 is one of the top 25 candidate genes identified in a microarray analysis of genes involved in EMT through regulation of Cdh1.^10^ Most importantly, one recent study has found that Grhl2 overexpression leads to increased metastatic potential in breast cancer.^11^ Cieply *et al.*,^12, 13^ however, reported that Grhl2 suppressed EMT, and also reported one feature of EMT, that is, the formation of large protrusive structures during the growth of colonies in three-dimensional Matrigel culture, indicative of invasive potential.^12^ In light of these studies, we hypothesized that Grhl2 may be closely related to cancer metastasis.

In our previous study, we have reported that Grhl2 functioned as a tumor suppressor in proliferation and apoptosis of gastric cancer.^14^ In the current study, we further explore the effect of Grhl2 on metastasis of gastric cancer as well as its possible molecular mechanisms.

## Results

### Grhl2 inhibits invasion and migration in both SGC7901 and MKN45 cells

**Figure 1:**
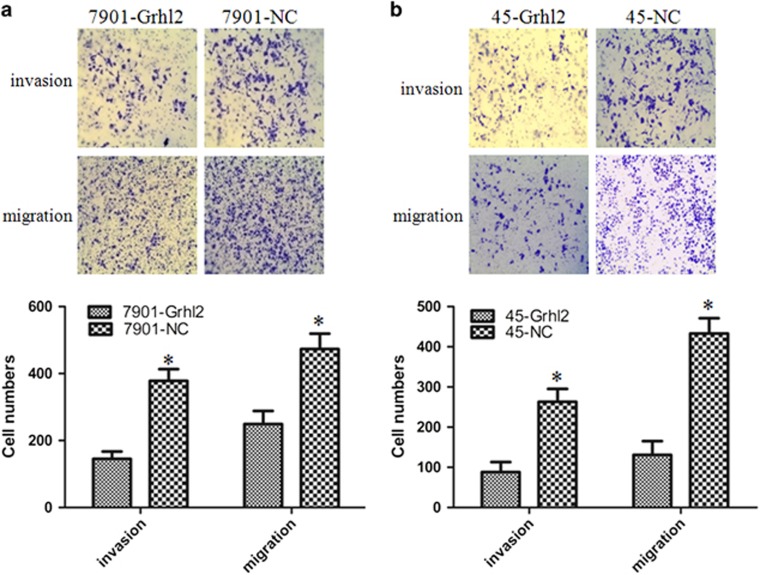
Grhl2 inhibits invasion and migration in both SGC7901 and MKN45 cells. (**a**) SGC7901 cells were infected with lentiviral-Grhl2-puro or lentiviral-control-puro, respectively. Overexpression of Grhl2 significantly inhibits the invasion and migration of SGC7901 cells. Representative images were provided. (**b**) Similar results were also observed in MKN45 cells. Original magnification × 100. *P<0.05.

In our previous study, we have proved that Grhl2 inhibits proliferation and promotes apoptosis so that it functioned as a tumor suppressor. Here we further investigate the effects of Grhl2 on invasion and migration of gastric cancer. Human gastric cancer cell lines SGC7901 and MKN45 were used in this study, and both the cell lines were infected with Grhl2 expression vector. Later, the establishment of stable cell lines are as described in the Materials and methods section. Invasion and migration assays were performed to detect the ability of metastasis *in vitro*. As a result, in invasion assays, we observed that cells that migrated through the membrane in 7901-Grhl2 groups were significantly less than that in 7901-NC groups (145±22 vs 378±35, *P*<0.05). In migration assays, the number of cells in 7901-Grhl2 groups was also significantly less than that in 7901-NC groups (249±39 vs 473±46, *P*<0.05) (Figure 1a). Analogical results were also seen in MKN45 cells (88±25 vs 263±32 and 131±34 vs 433±38 for invasion and migration, respectively, *P*<0.05) (Figure 1b). These results suggested that overexpression of Grhl2 can reduce the ability of invasion and migration of gastric cancer cells.

### Grhl2 reduces the expression of MMP-2, MMP-7 and MMP-9 in gastric cancer

**Figure 2:**
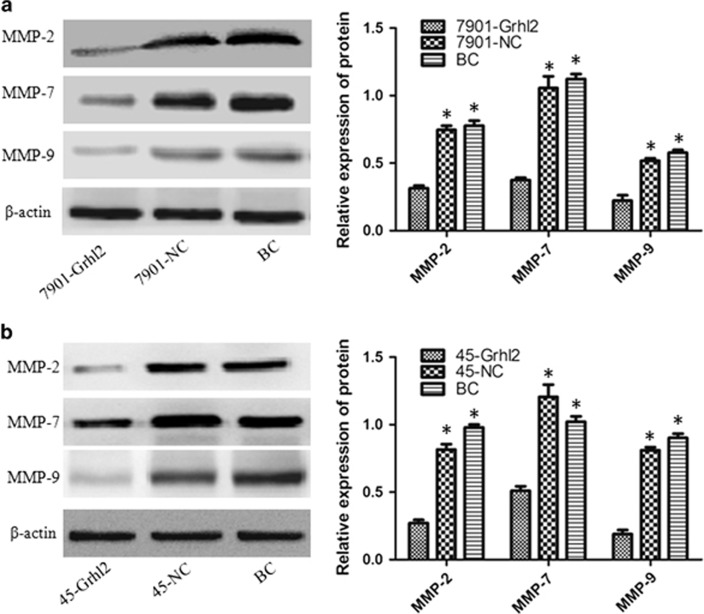
Grhl2 reduces the expression of MMP-2, MMP-7 and MMP-9 in gastric cancer. After Grhl2 overexpression in SGC7901 (**a**) and MKN45 (**b**) cells, the protein expression of MMP-2, MMP-7 and MMP-9 in each group was analyzed by western blot. β-Actin was used as an internal control in this study. BC, blank control; NC, negative control. *P<0.05.

According to the experimental results mentioned above, we verified the negative regulation of Grhl2 on invasion and migration of gastric cancer cells. To understand the possible mechanism of this phenomenon, changes of metastasis-associated protein were detected. Matrix metallopeptidase belong to a larger family of proteases that are capable of degrading all kinds of extracellular matrix proteins so as to play an important role in tumor metastasis. In this family, matrix metalloproteinase-2, -7 and -9 (MMP-2, MMP-7 and MMP-9) have been well studied.^15, 16, 17^ Western blot was performed to detect the protein expression of MMP-2, MMP-7 and MMP-9 in each group of SGC7901 and MKN45 cells. As shown in Figure 2, Grhl2 significantly reduced the expression of MMP-2, MMP-7 and MMP-9, compared with the negative and blank control group (*P*<0.05). However, no difference was observed between the negative control and blank control group. Importantly, the result was consistent between SGC7901 (Figure 2a) and MKN45 (Figure 2b) gastric cancer cells.

### Grhl2 antagonizes TGFβ-induced EMT in gastric cancer cells

**Figure 3:**
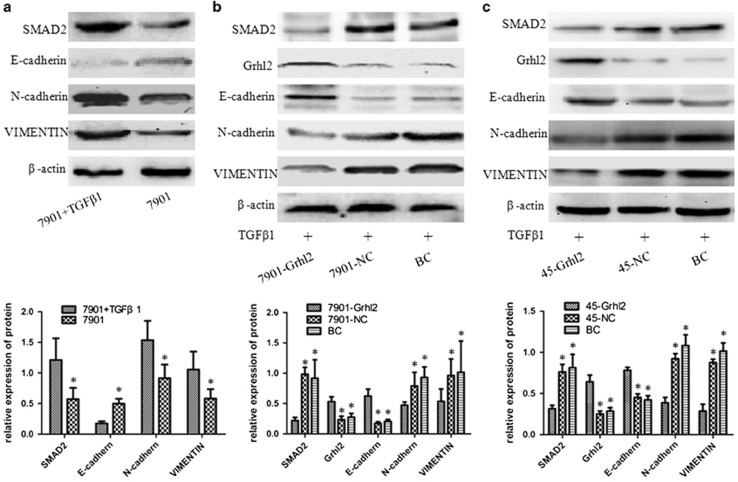
Grhl2 antagonizes TGFβ-induced EMT in SGC7901 and MKN45 cells. (**a**) SGC7901 cells were treated with a concentration of 2.5 ng/ml TGFβ1 for 24 h. Changes in SMAD2, E-cadherin, N-cadherin and Vimentin protein expression were detected by western blot. (**b**) The same concentration of TGFβ1 was added into the three groups of cells for 24 h, and protein expression of SMAD2, Grhl2, E-cadherin, N-cadherin and Vimentin were detected by western blot. (**c**) Same procedure was performed in MKN45 cells. *P<0.05.

Grhl2 reduces the expression of MMP-2, MMP-7 and MMP-9, which may partly explain the inhibitory effect of Grhl2 on invasion and migration of gastric cancer. However, the deep mechanisms by which Grhl2 inhibits the progression of gastric cancer were not clearly described. To better understand this issue, we investigated the effects of Grhl2 on TGFβ-induced EMT, which was closely related to tumor metastasis. TGFβ1, a common TGFβ signaling pathway agonist, was used for activating TGFβ signaling pathways in this study. First, SGC7901 cells were treated with a concentration of 2.5 ng/ml TGFβ1 for 24 h, an effective dose for the activation of TGFβ signaling pathways, as described elsewhere.^18^ As shown in Figure 3a, TGFβ1 significantly increased the expression of SMAD2, a key factor of TGFβ signaling pathways. TGFβ1 also increased N-cadherin and Vimentin expressions, but decreased E-cadherin levels. This result represents the activation of TGFβ signaling pathways and EMT process with a concentration of 2.5 ng/ml TGFβ1 in gastric cancer. Under this premise, we further analyzed alterations in the expression of EMT-associated protein as well as TGFβ signaling pathways in the case of with or without Grhl2 overexpression. As expected, TGFβ1 causes a striking upregulation of mesenchymal markers (N-cadherin and Vimentin) and a concomitant downregulation of epithelial marker (E-cadherin) in control groups. Although the same TGFβ1 stimulation, however, the expression of SMAD2, N-cadherin and Vimentin showed a striking reduction, whereas the expression of E-cadherin increased in 7901-Grhl2 groups compared with that in control groups (Figure 3b) (*P*<0.05). In addition, the results of MKN45 further confirmed this conclusion (Figure 3c). Taken together, these results strongly indicated that the effect of TGFβ-inducted EMT can be attenuated by Grhl2.

### Inhibition of TGFβ signaling pathways restores Grhl2 expression

**Figure 4:**
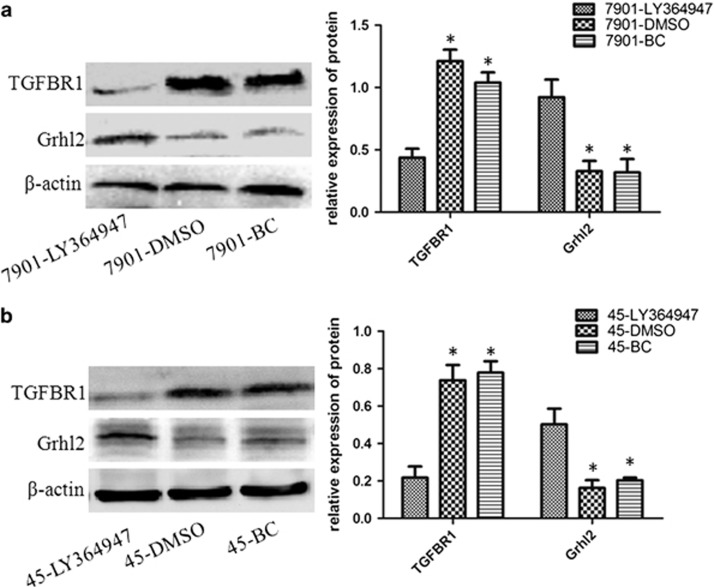
Inhibition of TGFβ signaling pathways restores Grhl2 expression. (**a**) SGC7901 cells were treated with a concentration of 400 nmol/l LY364947 for 24 h. Western blot was used to detect the protein expression of TGFβR1 and Grhl2. (**b**) Same procedure was performed in MKN45 cells. BC, blank control; DMSO, cells were treated with dimethyl sulfoxide; LY364947, cells were treated with LY364947. *P<0.05.

To further study the relationship between Grhl2 and TGFβ signaling pathways in the progress of gastric cancer. LY364947, an ATP-competitive and tight-binding inhibitor of TGFβR1, was used in the subsequent study. LY364947 with a concentration of 400 nmol/l was effective for inhibition of TGFβ signaling pathways.^19, 20^ Therefore, in the present study, cells were treated with 400 nmol/l LY364947. Cells were collected after 24 h and western blot was performed to detect the expression of TGFβR1 and Grhl2. As in Figure 4, LY364947 downregulated TGFβR1 expression and is accompanied by increased expression of Grhl2 in both SGC7901 (Figure 4a) and MKN45 (Figure 4b) cells. This result is consistent with that obtained by Cieply *et al.*^12^ and revealed the reciprocal relationship between Grhl2 and TGFβ signaling pathways during tumorigenesis. Thus, we concluded that downregulation of Grhl2 in gastric cancer may, at least in part, be caused by TGFβ signaling pathways. Most importantly, this is a reversible process.

### Grhl2 inhibits gastric cancer growth and metastasis *in vivo*

**Figure 5:**
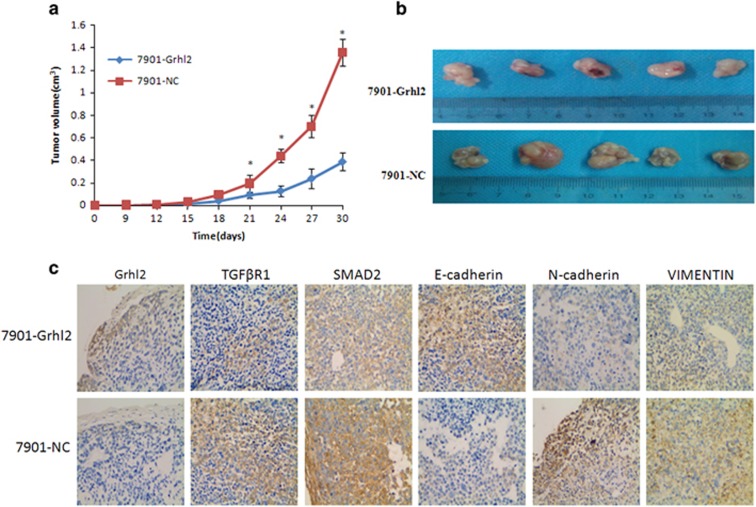
Grhl2 inhibits gastric cancer growth and reverses EMT process *in vivo*. (**a**) The nude mice were injected with 7901-Grhl2 or 7901-NC, respectively. Tumor size was measured every 3 days. Growth curves were evaluated with time points as the abscissa and tumor volumes as the vertical coordinate. (**b**) After 30 days, the mice were killed and tumor samples were taken. (**c**) Tumor samples were used to perform immunohistochemistry for analyzing the protein expression of Grhl2, SMAD2, TGFβR1, E-cadherin, N-cadherin and Vimentin. *P<0.05.

To further validate the inhibitory role of Grhl2 in gastric cancer, subcutaneous xenograft model was used for testing the effects of Grhl2 on tumor growth *in vivo*. Xenografts were observed at 9 days after the cells were administered into the nude mice. The result showed that tumor growth in 7901-Grhl2 groups is significantly slower compared with that in control groups (Figure 5a and Table 1). After 30 days, as expected, the tumor volume was significantly smaller in 7901-Grhl2 groups compared with that in control groups (Figure 5b). Later, immunohistochemistry was performed to detect Grhl2-, TGFβR1-, SMAD2- and EMT-associated protein expression in tumors. We found that Grhl2 and the epithelial marker (E-cadherin) increased, while TGFβR1, SMAD2 and mesenchymal markers (N-cadherin and Vimentin) significantly decreased, in the 7901-Grhl2 group compared with that in control groups (Figure 5c). These results together indicated that Grhl2 inhibits gastric cancer growth and reverses EMT process *in vivo*.

**Figure 6:**
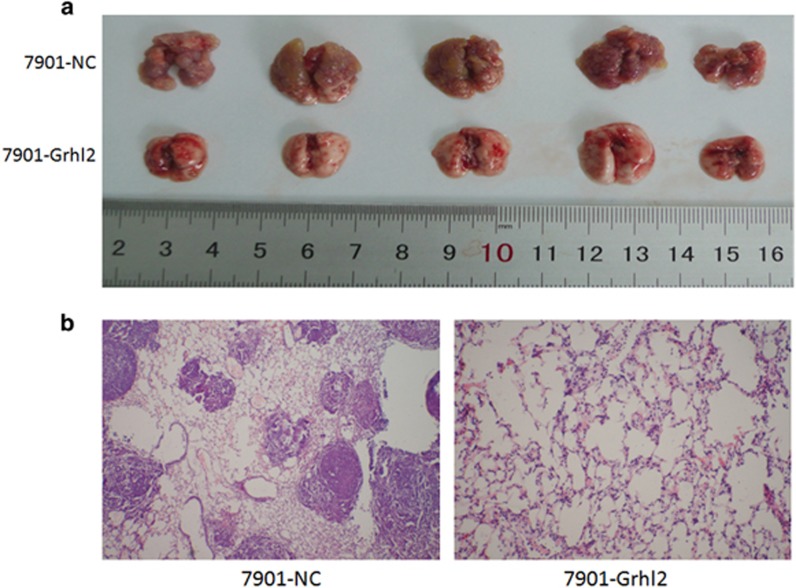
Grhl2 inhibits metastasis of gastric cancer *in vivo*. (**a**) Mice were treated with 7901-Grhl2 or 7901-NC, respectively. After 4 weeks, lung tissues were isolated to observe the metastatic tumors. (**b**) Furthermore, tissues were sectioned serially and then stained with hemtoxylin and eosin (H&E).

On the other hand, we constructed an *in vivo* metastatic model to study the effect of Grhl2 on the metastatic potential of gastric cancer cells. Mice were divided into two groups, and 7901-Grhl2 and 7901-NC were injected into the tail vein, respectively. After 4 weeks, liver and lung tissues were isolated. As a result, despite we did not find obvious lesions in the liver (data not show), the 7901-Grhl2 group had significantly less metastatic tumors on the surface of the lungs compared with that of the 7901-NC group (Figure 6a) (*P*<0.05). Then, hemtoxylin and eosin staining were performed to detect the lungs of the two groups (Figure 6b). Thus, our results demonstrated that Grhl2 suppresses the metastasis of gastric cancer *in vivo*.

## Discussion

Gastric cancer is a common digestive system malignant tumor seriously affecting the human health. However, high degree of malignant but limited treatment options makes the prognosis of patients with gastric cancer still not satisfactory. Metastasis is one of the most important factor contributing to cancer-related mortality. It is generally thought that effective control of metastasis can significantly reduce mortality. In view of this, in this study, we aim to investigate the underlying mechanisms of invasion and migration in the hope of providing new strategy for the diagnosis and treatment of gastric cancer.

In our previous study, Grhl2 was identified as a tumor suppressor because of its role on proliferation and apoptosis of gastric cancer.^14^ Here we further defined the inhibitory effect of Grhl2 on gastric cancer invasion and migration ability. These results together indicated that Grhl2 functioned as a tumor suppressor but downregulated in gastric cancer. This conclusion may be contradictory with some other reports, which suggested that Grhl2 may be an oncogene.^11, 21, 22, 23, 24, 25^ The reasons for this discrepancy have been explained in our previous study.^14^ In spite of this, however, it is important to note that Cieply *et al.*^12^ have reported that Grhl2 is predicted to act as a tumor suppressor gene (i.e., in EMT-like subclasses). Moreover, Werner *et al.*^26^ directly pointed out the dual roles of Grhl2 in breast cancer. Furthermore, in our preliminary experiments, high expression of Grhl2 was also seen in NCI-N87 gastric cancer cells, which represent a kind of high differentiation of gastric cancer cell line (data not show). Thus, we hypothesized that the degree of differentiation may be an another factor that determines Grhl2 expression. This issue will be investigated in the next experiment.

MMPs family is thought to be involved in multiple pathways including its role in metastasis. Therefore, we tested the changes of MMP-2, MMP-7 and MMP-9 after Grhl2 overexpression in both SGC7901 and MKN45, so as to preliminarily clear the possible mechanisms by which Grhl2 inhibits invasion and migration of gastric cancer. However, how Grhl2 reduces MMP-2, MMP-7 and MMP-9 expression is another interesting issue and needs further research. In the present study, Grhl2 reduces MMP-2, MMP-7 and MMP-9 expression, which is partly explained by the mechanism by which Grhl2 inhibits gastric cancer cell invasion and migration, but this is obviously not enough. However, deeper mechanisms need to be explored.

EMT is a highly conserved developmental program that allows polarized, immotile epithelial cells to convert to those with motile mesenchymal properties.^27^ Recently, accumulating data indicated that EMT has an important role in tumor metastasis.^4, 28, 29^ Most importantly, Xiang *et al.*^11^ reported that during TGFβ-induced EMT, Grhl2 is downregulated and Grhl2 is always downregulated in cells that had undergone EMT. Cieply *et al.*^12^ revealed that Grhl2 suppresses TGFβ-induced EMT in breast cancer. Therefore, we questioned whether the same molecular mechanism is also effective in gastric cancer. In this study, our results demonstrated that TGFβ-induced EMT can be abrogated by overexpression of Grhl2 in gastric cancer. Moreover, inhibition of TGFβ signaling pathway can restore Grhl2 expression in gastric cancer. Finally, subcutaneous xenograft model further validated the growth inhibition and EMT reversal of Grhl2 on gastric cancer *in vivo*. Moreover, metastatic model indicated that Grhl2 significantly inhibits the metastatic potential of gastric cancer. These results commonly proved the inhibitory effect of Grhl2 on gastric cancer *in vivo* and *in vitro*. Meanwhile, we reveal the relationship between Grhl2 and TGFβ signaling pathways in gastric cancer. Thereby, to a certain extent, clarifying that a systematic mechanism of Grhl2 suppresses the progression of gastric cancer.

Since Xiang *et al.*^11^ reported that downregulation of Grhl2 could be a necessary step during EMT. Which one is the first step in gastric cancer progression (Grhl2 downregulation or EMT process)? This is an intriguing question and relates to whether Grhl2 could be seen as a predictor in early gastric cancer. In addition, there are several articles reported that GRHL2 and ZEB1 transcription factors form a negative feedback loop.^12, 13, 24, 26^ Thus, are there any other targets of Grhl2 in TGFβ signaling pathways? We look forward to the answers of these questions in the next experiments.

In conclusion, our study identified Grhl2 as a tumor suppressor, through regulation of TGFβ signaling pathways, involved in the occurrence and development of gastric cancer. It may provide a new strategy for the clinical diagnosis and treatment of gastric cancer.

## Materials and methods

### Materials

The lentiviral-Grhl2-puro and lentiviral-control-puro were purchased from SunBio (Shanghai, China). Anti-Grhl2 was purchased from Abcam Biotechnology (Cambridge, UK). Anti-MMP-2, MMP-7, MMP-9, SMAD2 and β-actin were from Proteintech Group Inc. (Wuhan, China). Anti-E-cadherin was purchased from Cell Signaling Technology (Beverly, MA, USA). Anti-Vimentin, TGFβR1 and N-cadherin were obtained from Epitomics Inc. (Burlingame, CA, USA). LY364947 and TGFβ1 were purchased from Sigma-Aldrich (St Louis, MO, USA).

### Cell culture

Human gastric carcinoma cell line SGC7901 was purchased from the cell bank of Chinese Academy of Sciences (Beijing, China). Human gastric carcinoma cell lines MKN45 was obtained from the Key Laboratory of General Surgery in Chongqing. The cells were cultured in RPMI-1640 medium (Hyclone, Beijing, China) containing 10% fetal bovine serum (Hyclone). The temperature and CO_2_ concentration were kept at 37 °C and 5%, respectively.

### Establishment of lentiviral infection and stable cell lines

Establishment of lentiviral infection and stable cell lines were described in our previous study.^14^ Briefly, SGC7901 and MKN45 cells were infected with lentiviral-Grhl2-puro (designated as 7901-Grhl2 and 45-Grhl2, respectively) or lentiviral-control-puro (designated as 7901-NC and 45-NC, respectively) at a multiplicity of infection of 30. Stable cell lines were generated by puromycin dihydrochloride (5 μg/ml; SunBio, Beijing, China) selection for 7 days.

### Invasion and migration assays

The 8.0 μm pore size Transwell chambers (Millicel; Millipore, Bedford, MA, USA) were used for invasion and migration assays. For invasion assays, Transwell chambers were coated with Matrigel (dilution 1:4, BD Biosciences, San Jose, CA, USA). After solidification, cells (1 × 10^5^) were seeded on upper Transwell chamber insert in 24-well plates and cultured in RPMI-1640 medium supplemented with 1% FBS. RPMI-1640 medium supplemented with 10% FBS was added to the 24-well plates. After 24 h, non-invading cells on the upper surface of membranes were wiped out by cotton swab and those on the underside were stained with crystal violet and counted under a light microscope with a magnification of × 100. Similar methods were performed for migration assays, except that Matrigel was not used.

### Western blot analysis

The detailed steps for electrophoresis, transfer and immunoblotting were described previously.^14^ Antibodies used in this study were mentioned in the Materials and methods section. Each experiment was repeated three times, and the images were analyzed using Quantity One (Bio-Rad, Hercules, CA, USA).

### Animal studies

Five-week-old male BALB/c nude mice were purchased from the Laboratory Animal Center of Chongqing Medical University (Chongqing, China). The study was approved by the Medical Ethics Review Committee of First Affiliated Hospital of Chongqing Medical University. For subcutaneous xenograft model, mice were randomly divided into two groups (five mice per group) and were injected with 2 × 10^6^ cells (7901-Grhl2 or 7901-NC) in the flanks of nude mice. Tumor size was measured every 3 days. After 30 days, the mice were killed and tumor samples were taken. Tumor volumes were calculated according to the method as described elsewhere.^30^ Growth curves were evaluated with time points as the abscissa and tumor volumes as the vertical coordinate.

Metastatic model was constructed as follows: mice were randomly divided into two groups (five mice per group). A total of 2 × 10^6^ 7901-Grhl2 or 7901-NC cells in 200 μl phosphate-buffered saline were injected into the tail vein, respectively. The mice were killed 4 weeks later, and the lung and liver tissues were collected for metastasis analysis.

### Immunohistochemistry

The detailed steps for antigen retrieval, blocking and antibody incubation were described previously.^14^ The results were assessed by two independent investigators. Antibodies used in this study were mentioned in the Materials and methods section. The staining result was analyzed as described elsewhere.^31^

### Statistical analysis

All data were analyzed using SPSS 17.0 (SPSS Inc, Chicago, IL, USA). Data are presented as mean±s.e.m. The Student's two-tailed *t*-test was used to assess statistically significant differences between experimental and control groups. Analysis of variance was used when more than two data sets were analyzed, followed by an appropriate *post hoc* test. *P*<0.05 was considered statistically significant.

## Figures and Tables

**Table 1:** Tumor volume at different time points

*Time/groups*	*0 day*	*9 days*	*12 days*	*15 days*	*18 days*	*21 days*	*24 days*	*27 days*	*30 days*
7901-Grhl2	0±0	0.0032±0	0.0064 ±0	0.0192±0	0.0411±0.01	0.0919±0.03	0.1283±0.05	0.2385±0.09	0.3892±0.08
7901-NC	0±0	0.0032±0	0.0064±0	0.0324±0	0.0936±0.01	0.1938±0.08	0.4389±0.06	0.7007±0.1	1.3599±0.12

Abbreviation: Grhl, grainyhead-like 2.
